# Safety and Efficacy of Acute Clopidogrel Load in Patients with Moderate and Severe Ischemic Strokes

**DOI:** 10.1155/2016/8915764

**Published:** 2016-10-13

**Authors:** Amir Shaban, Dominique J. Monlezun, Natalia Rincon, Jonathan Tiu, Melisa Valmoria, Sheryl Martin-Schild

**Affiliations:** ^1^Department of Neurology, Tulane University School of Medicine, New Orleans, LA, USA; ^2^Department of Neurology, University of Iowa Hospitals and Clinics, Iowa City, IA, USA; ^3^Department of Global Health Management and Policy, Tulane University School of Public Health, New Orleans, LA, USA; ^4^University of South Carolina School of Medicine, Greenville, SC, USA; ^5^Stroke Program, Department of Neurology, Tulane University School of Medicine, New Orleans, LA, USA

## Abstract

*Objective*. To study the safety and efficacy of a clopidogrel loading dose in patients with moderate and severe acute ischemic strokes.* Background*. The safety of clopidogrel loading has been extensively investigated in patients with minor strokes and transient ischemic attacks.* Methods*. Acute ischemic stroke patients presenting consecutively to our center from 07/01/08 to 07/31/13 were screened. Clopidogrel loading was defined as at least 300 mg dose (with or without aspirin) given within 6 hours of admission. We compared outcomes in patients with baseline NIHSS > 3 with and without clopidogrel loading.* Results*. Inclusion criteria were met for 1011 patients (43.6% females, 69.1% black, median age 63). Patients with clopidogrel loading had lower baseline NIHSS than patients who were not loaded (8 versus 9, *p* = 0.005). The two groups had similar risk for hemorrhagic transformation (*p* = 0.918) and symptomatic hemorrhage (*p* = 0.599). Patients who were loaded had a lower rate of neurological worsening (38.9% versus 48.3%, *p* = 0.031) and less in-hospital mortality (4.3% versus 13.4%, *p* = 0.001) compared to those who were not loaded. The likelihood of having a poor functional outcome did not differ between the two groups after adjusting for NIHSS on admission (OR = 0.71, 95% CI 0.4633–1.0906, *p* = 0.118).* Conclusion*. Clopidogrel loading dose was not associated with increased risk for hemorrhagic transformation or symptomatic intracranial hemorrhage in our retrospective study and was associated with reduced rates of neuroworsening following moderate and severe stroke.

## 1. Introduction

Clopidogrel is commonly used for ischemic stroke prophylaxis. Several studies proposed a method for using clopidogrel by administrating a loading dose acutely in the setting of ischemic stroke patients who are not eligible for tPA treatment and who are at risk of progressive stroke. While nonloading doses of clopidogrel require 3 to 5 days for full inhibition of platelet aggregation, loading doses of 300 mg of clopidogrel show significant inhibition within 6 hours [1, 2]. Several major clinical trials studied the safety and efficacy of clopidogrel loading in minor acute ischemic strokes (AIS) and transient ischemic attacks (TIA). Patients with mild AIS and TIAs have been known for being at a high risk of recurrent strokes [3–5]. These patients are frequently excluded from tPA therapy because their deficits are too mild for treatment, have completely resolved by the time they arrive to the hospital, or are presenting outside the window for tPA therapy [6]. For those reasons clinical trials limited enrollment to minor AIS and TIAs [7–9]. As a result of that limitation, only scarce data are available in the literature with regard to the safety and efficacy of clopidogrel loading therapy in patients with more than minor ischemic strokes.

In this study we investigated the safety and efficacy of using a clopidogrel loading dose in moderate and severe ischemic strokes by examining bleeding complications and neuroworsening due to stroke progression.

## 2. Methods

All patients who consecutively presented to the Stroke Service at Tulane Medical Center with AIS between 07/2008 and 10/2013 were identified from our prospectively collected stroke registry [10]. Patients younger than 18 years of age and patients who received tPA were excluded from all analyses.

Stroke etiology was classified according to Trial of Org in Acute Stroke Treatment [11]. Clopidogrel loading dose of at least 300 mg (with or without aspirin) was given within 6 hours of admission when tPA was contraindicated and patients were at risk of progressive stroke without completed full territorial infarction on baseline imaging. Of note previous studies varied in the time of clopidogrel load administration (36, 24, and 12 hours) after stroke onset with more recent studies striving to administrate clopidogrel as early as possible [7–9, 12].

### 2.1. Definitions

Patients with (NIHSS ≥ 4) were considered to have moderate to severe strokes [3–5]. Symptomatic intracranial hemorrhage (sICH) was defined as a parenchymal hematoma associated with an increase in NIHSS by at least 4 points. Inpatient complications were defined as an incident of infections, angioedema, systemic bleeding, or recurrent thrombotic events.

Poor functional outcome was defined as mRS > 2 on discharge. Neuroworsening was defined as an increase in NIHSS by at least 2 points within any 24-hour period [13]. Unfavorable discharge was defined as disposition other than home or inpatient rehabilitation.

### 2.2. Statistics

The first set of analyses was performed on all patients who received clopidogrel loading dose. We compared patients who had minor AIS to those who had more than minor AIS. In the second set only patients with more than minor AIS were analyzed.

Pearson's chi-square or Fisher's exact test was used to compare categorical data. Independent samples *t*-test or Wilcoxon rank-sum test was used to compare continuous data, where appropriate.

Multivariate regression analyses were conducted, both with and without adjustment for age, glucose level on admission, and baseline NIHSS. Missing cases were censored during analysis.

## 3. Results

Inclusion criteria were met in 1011 patients; of these, 43.6% (441/1011) were women and 69.1% (699/1011) were Black, median age 63. Of the included patients, 365/1011 were loaded with clopidogrel.

### 3.1. Loading Mild Strokes versus Loading Moderate and Severe Strokes

Of 365 patients who were loaded, 155 (42.5%) patients had mild ischemic stroke (NIHSS < 4) and 209 (57.3%) patients had more than mild ischemic strokes (NIHSS ≥ 4).  Table 1 demonstrates the demographics of the two groups (Table 1).

Patients with mild strokes had shorter hospital stay and lower complication rate (Table 1).

Patients with mild strokes were less likely to have a hemorrhagic transformation, 3 (5.8%), compared to patients with moderate to severe strokes, 24 (24.24%), *p* = 0.006. When adjusted to age and glucose levels on admission, patients with moderate and severe strokes were 7 times more likely to have a new hemorrhage or hemorrhagic transformation during admission (OR = 7.6; CI 1.49, 18.30; *p* = 0.003) than patients with mild strokes. The two groups had similar frequencies of sICH, *p* = 1.000.

### 3.2. Loading versus Not Loading Moderate and Severe Strokes

Of 1011 ischemic stroke patients included in the study 590 (58.4%) had moderate to severe ischemic stroke. Of these 209 (35.4%) were loaded. There was no difference between patients who were loaded and those who were not in terms of stroke risk profile (Table 2).

Patients who were loaded had lower NIHSS at baseline than those who were not loaded (median NIHSS 8 versus 9, *p* = 0.005). Patients who were loaded had shorter hospital length of stay compared to those who were not loaded (median length of stay 7 versus 8 days, *p* = 0.001). Patients who were loaded were significantly less likely to experience neuroworsening, 81 (38.9%), compared to patients who did not receive clopidogrel load, 179 (48.3%), *p* = 0.031. Statistical significance was lost after adjusting to NIHSS on admission. Patients who were loaded had significantly less in-hospital mortality, 9 (4.3%), compared to those who were not loaded, 51/381 (13.4%), *p* = 0.001. There was no significant difference in terms of inpatient complications between the two groups, *p* = 0.105. Patients who were loaded had significantly better functional outcomes (median mRS 3 versus 4, *p* < 0.001) and significantly less NIHSS on discharge (median NIHSS 4 versus 6, *p* = 0.002) than those who were not loaded (Table 2). Poor functional outcomes were more frequent in patients who were not loaded (81.6% versus 72.7%, *p* = 0.012). Statistical significance was lost after adjusting to age, glucose, NIHSS on admission, HTN, HLD, DM, and smoking, *p* = 0.079.

Patients who received clopidogrel load did not have a higher risk of hemorrhagic transformation, 24 (24.4%), compared to those who did not, 56 (24.8%), *p* = 0.918. Patients who were loaded with clopidogrel did not have a higher risk of sICH, 2 (1.8%), compared to those who were not, 2 (0.9%), *p* = 0.599. The risk for hemorrhagic transformation and sICH was similar between the two groups after adjusting to age, glucose, NIHSS on admission, HTN, HLD, DM, and smoking, *p* = 0.834. This remained insignificant after adjusting to dual antiplatelet use of aspirin and Plavix on admission (Table 3).

## 4. Discussion

Frequently stroke specialists find themselves in a situation where tPA is contraindicated and often clopidogrel loading is the chosen intervention as an attempt to stop stroke progression. There is currently good evidence that loading with clopidogrel does not increase the risk of bleeding in patients with minor AIS or TIAs [7–9].

Often clopidogrel is given to patients with moderate to severe strokes despite the absence of strong data to support this practice.

It has been established that the risk of hemorrhagic transformation or sICH after treatment with tPA is increased as the stroke severity (NIHSS) increases [14, 15]. Is that the case for clopidogrel loading as well? Only scarce data is available on the safety and efficacy of this therapy in patients with moderate and severe strokes. A previous pilot study (*n* = 40, mean NIHSS = 6) suggested that loading with clopidogrel within 36 hours of symptom onset was safe; the study did not stratify the safety based on the stroke severities [12]. Another study (*n* = 341, median NIHSS = 4) showed that loading with clopidogrel was not associated with short-term increase in bleeding events [16].

### 4.1. Loading Mild versus Loading Moderate and Severe Strokes

In our study patients with moderate and severe stokes were more likely to have a hemorrhagic transformation after receiving clopidogrel load compared to those with mild strokes. Although this finding may easily be explained by the differences in the stroke severity at presentation, we conducted this comparison because, in the absence of strong data to guide the use of clopidogrel loading in patients with moderate and severe stroke, our finding of higher rates of hemorrhagic transformation may create an illusion that clopidogrel loading is driving the hemorrhagic transformation and deter providers from loading with clopidogrel in these patients. To further investigate that matter we conducted a second analysis on patients with moderate to severe strokes to compare those who received a clopidogrel loading dose to those who did not.

### 4.2. Loading versus Not Loading Moderate to Severe Strokes

Patients with moderate to severe strokes who were loaded did not have a higher frequency of hemorrhagic transformation than those who were not loaded. In our study the administration of clopidogrel loading doses to moderate and severe strokes did not increase the risk of hemorrhagic transformation or sICH during the hospital admission. In the absence of clinical trials proving the safety of clopidogrel loading in patients with moderate to severe strokes, the decision to load or not to load is left to the discretion of the treating specialist. We hope that our data will help make a more informed decision and will provide a platform for future clinical trials.

In our study patients who were loaded with clopidogrel had less neuroworsening and shorter length of stay; however, there was no significant difference in other short-term outcome measures.

### 4.3. Limitations

The limitations include the retrospective nature and the absence of randomization in choosing who will be loaded. Repeated brain imaging was not performed routinely; therefore, sampling bias likely limits examination of brain bleeding to those who experienced neuroworsening. The true rate of asymptomatic hemorrhagic infarction after clopidogrel loading is, for this reason, unknown. Patients with moderate and severe strokes who received clopidogrel load had less NIHSS on admission compared to patients with moderate and severe strokes who were not loaded; this makes some of the study results difficult to interpret. Patients who received dual antiplatelet therapy on admission did not necessarily stay on the dual antiplatelet for the rest of their admission.

## Figures and Tables

**Table 1:** Demographic and outcome variables according to NIHSS score and load status.

	NIHSS 3 or less and loaded	NIHSS 4 or more and loaded	*p* value
	*n* = 155 (42.6%)	*n* = 209 (57.4%)	
Age, median years (min–max)	61 (21–90)	65 (31–99)	<0.001
African American, number (%)	102/155 (65.8%)	151/209 (72.3%)	0.341
Gender, number of females (%)	67/155 (43.2%)	98/209 (46.9%)	0.487
Hyperlipidemia	66/155 (42.6%)	95/208 (45.7%)	0.557
HTN	119/155 (76.8%)	174/208 (83.6%)	0.100
Smoking	63/154 (40.9%)	78/205 (38.1%)	0.583
DM	55/153 (35.95%)	74/204 (36.27%)	0.949
Prior stroke	50/155 (32.26%)	107/209 (51.20%)	<0.001
Daily alcohol	12/81 (14.81%)	16/106 (15.09%)	0.958
Glucose on admission	108 (68–495)	116 (57–574)	0.199
Atrial fibrillation	6 (3.9%)	21 (10.5%)	0.022
Dual Plavix and aspirin	141 (92.2%)	184 (91.5%)	0.835
Length of stay, median (min–max)	3 (1–39)	7 (1–52)	<0.001
Inpatient complications	11/87 (12.7%)	54/137 (39.4%)	<0.001
UTI	5/155 (3.2%)	31/209 (14.8%)	<0.001
Pneumonia	2/155 (1.3%)	17/208 (8.2%)	0.004
Recurrent thrombotic events	29/153 (19.0%)	86/206 (41.8%)	<0.001
Neuroworsening%	26/153 (17.0%)	81/208 (38.9%)	<0.001
TOAST			0.342
Cardioembolic	27/155 (17.4%)	49/209 (23.4%)	
Large vessel	36/155 (23.2%)	57/209 (27.3%)	
Small vessel	50/155 (932.3%)	56/209 (26.8%)	
Cryptogenic > 1 cause	5/155 (3.2%)	3/209 (1.4%)	
Cryptogenic no cause	25/155 (16.1%)	25/209 (12.0%)	
Other (no vessel dissection)	12/155 (7.7%)	19/209 (9.1%)	
Hemorrhagic infarct in 36 h on F/uCT1 or F/uCT2	3/52 (5.8%)	24/99 (24.2%)	0.006
Symptomatic hemorrhage F/uCT1 or F/uCT2	1/60 (1.7%)	2/114 (1.8%)	1.000

**Table 2:** Demographic and outcome variables according to NIHSS score and load status.

	NIHSS ≥ 4, not loaded	NIHSS ≥ 4, loaded	*p* value
Age, median years (min–max)	65 (19–103)	65 (31–99)	0.989
African American, number (%)	269 (70.6%)	151 (72.3%)	0.757
Gender, number of females (%)	184 (48.3%)	98 (46.9%)	0.744
Hyperlipidemia	148 (39.2%)	95 (45.7%)	0.125
HTN	294 (77.6%)	174 (83.7%)	0.080
Smoking	121 (31.8%)	78 (38.1%)	0.131
DM	141 (37.9%)	74 (36.3%)	0.699
Prior stroke	179 (47.2%)	107 (51.2%)	0.357
Glucose on admission	124 (61–831)	116 (57–574)	0.207
Atrial fibrillation	59 (15.7%)	21 (10.1%)	0.060
Dual Plavix and aspirin	0 (0.0%)	184 (91.5%)	<0.001
Admission NIHSS, median (min–max)	9 (4–37)	8 (4–31)	0.005
Length of stay, median (min–max)	8 (0–95)	7 (1–52)	0.001
Inpatient complications	139/291 (47.8%)	54/137 (39.4%)	0.105
UTI	67 (17.6%)	31 (14.8%)	0.383
Pneumonia	42 (11.0%)	17 (8.2%)	0.271
Recurrent thrombotic events	190 (51.5%)	86 (41.8%)	0.025
Neuroworsening%	179 (48.3%)	81 (38.9%)	0.031
Poor functional outcome (mRS > 2)	311 (81.6%)	152 (72.7%)	0.012
Unfavorable disposition	29 (10.9%)	11 (16.9%)	0.178
NIHSS d/c	6 (0–42)	4 (0–42)	0.002
mRS discharge, median (min–max)	4 (0–6)	3 (0–6)	<0.001
mRS 0–2	70 (18.4%)	57 (27.3%)	0.012
mRS 3-4	200 (52.5%)	114 (54.6%)	0.633
mRS 5-6	111 (29.1%)	38 (18.2%)	0.003
Hemorrhagic infarct in 36 h on F/uCT1 or F/uCT2	56 (24.8%)	24 (24.2%)	0.918
Symptomatic hemorrhage F/uCT1 or F/uCT2	2 (0.9%)	2 (1.8%)	0.599

**Table 3:** Odds ratios.

Predictor	Outcome	Unadjusted	Adjusted^*∗*^	Fully adjusted^*∗∗*^	Fully adjusted^*∗∗∗*^
Loaded and NIHSS > 3 versus not loaded and NIHSS > 3	Poor functional outcome	0.60 (0.402–0.90) *p* value = 0.012	0.56 (0.366–0.85) *p* value = 0.006	0.67 (0.42–1.06) *p* value = 0.087	2.97 (0.36–24.40) *p* value = 0.310
Loaded and NIHSS > 3 versus not loaded and NIHSS > 3	Unfavorable discharge disposition	0.71 (0.50–1.02) *p* value = 0.061	0.70 (0.49–1.00) *p* value = 0.050	0.85 (0.57–1.26) *p* value = 0.423	1.77 (0.59–5.30) *p* value = 0.307
Loaded and NIHSS > 3 versus not loaded and NIHSS > 3	New infarct during admission	1.18 (0.41–3.41) *p* value = 0.764	1.28 (0.43–3.74) *p* value = 0.657	1.59 (0.48–5.33) *p* value = 0.448	0.60 (0.05–6.74) *p* value = 0.677
Loaded and NIHSS > 3 versus not loaded and NIHSS > 3	New hemorrhage or hemorrhagic infarct during admission	0.97 (0.56–1.68) *p* value = 0.918	1.00 (0.57–1.73) *p* value = 0.995	1.27 (0.71–2.27) *p* value = 0.420	0.0 (0-0) *p* value = 0.989

^*∗*^Adjusted for age, glucose admin; ^*∗∗*^adjusted for age, NIHSS baseline, glucose admin, HTN, HLD, DM, and smoking; ^*∗∗∗*^adjusted for age, NIHSS baseline, glucose admin, HTN, HLD, DM, smoking, and dual therapy.
